# Enhanced Recovery After Liver Surgery: Does Compliance Impact Survival?

**DOI:** 10.1245/s10434-026-19459-7

**Published:** 2026-04-02

**Authors:** Martina Mariatti, Francesca Venza, Alessandra Cristaudi, Lorenzo Bernardi, Isabella Hunjan, Paul Boulard, Fabrice Muscari, Eddy Cotte, Guillaume Passot, Pascale Mariani, Rabih-Tany Mikhael, Olivier Detry, Gabriel Thierry, Benjamin Muller, Olivier Facy, Aurélien Dupre, Nicolas Mouton, David-Jérémie Birnbaum, Théophile Guilbaud, Giuliana Amaddeo, Paul Vigneron, Daniele Sommacale, Karem Slim, Alexandre Doussot, Raffaele Brustia

**Affiliations:** 1 INSERM U955, Team ‘‘Pathophysiology and Therapy of Chronic Viral Hepatitis and Related Cancers’’, Créteil, France; 2 Department of Digestive and Hepato-pancreatic-biliary Surgery, AP-HP, Hôpital Henri-Mondor, Assistance Publique-Hôpitaux de Paris, GRCT OPTIMA, Créteil, France; 3 Paris Est Créteil University, UPEC, Créteil, France; 4 Digestive Surgical Oncology - Liver Transplantation Unit, National Referral Center-Echninococcosis. CNR-E, CHU Besancon, France, CHU Besancon, France; 5 Division of Hepato-Pancreato-Biliary Surgery, Department of Surgery, Ente Ospedaliero Cantonale and Faculty of Biomedical Sciences, University of Southern Switzerland, Lugano, Switzerland; 6 Digestive Surgery and Transplantation Department, Toulouse University Hospital Centre, Toulouse, France; 7 Service de chirurgie digestive et oncologique, Hôpital Lyon-Sud - CHU de Lyon, Lyon, France; 8 Department of Surgical Oncology, Institut Curie, PSL Research University, Paris, France; 9 Department of Abdominal Surgery and Transplantation, CHU Liege, University of Liege (CHU ULg), Liege, Belgium; 10 Department of Anesthesia and Intensive Care Medicine, CHU Liege, University of Liege (CHU-ULg), Liege, Belgium; 11 Digestive, Endocrine and Cancer Surgery Department, CHU Dijon, Dijon, France; 12 Department of Surgical Oncology, Inserm, U1032, LabTau, Lyon, Centre Léon Bérard, Lyon, France; 13 Univ Lyon, Lyon, France; 14 Digestive and Oncological Surgery Unit, Hôpital Nord, Assistance Publique Hôpitaux de Marseille, Faculté de Médecine de Marseille, Aix-Marseille Université, Marseille, France; 15 INSERM U955, Team ‘‘Pathophysiology and Therapy of Chronic Viral Hepatitis and Related Cancers’’, Créteil, France; 16 Department of Hepatology, AP-HP, Hôpital Henri-Mondor, Assistance Publique-Hôpitaux de Paris, Créteil, France; 17 Paris Est Créteil University, UPEC, Créteil, France; 18 Pôle Santé République (ELSAN Group), Clermont-Ferrand, France; 19 Francophone Group for Enhanced Recovery After Surgery (GRACE), Beaumont, France

**Keywords:** Enhanced Recovery Protocol, Liver surgery, Multicenter prospective cohort, Survival

## Abstract

**Background:**

Enhanced recovery programs (ERPs) improve short-term outcomes after liver surgery (LS), but their impact on survival remains uncertain. This study evaluated the association between ERP compliance and survival in a large multicenter cohort.

**Methods:**

This prospective European multicenter cohort included adults undergoing elective LS between 2016 and 2024 in 10 centers implementing Enhanced Recovery After Surgery (ERAS)-consistent ERPs. Compliance was defined as the proportion of 21 fulfilled items with high compliance (≥70%). A 1:1 propensity score-matching was performed to balance baseline characteristics. The primary outcome was 12 month overall survival (OS). Secondary outcomes included predictors of 12 month and long-term OS and cancer-specific subgroup analyses. Cox regression models, Kaplan–Meier analyses, and sensitivity analyses including center effects were used.

**Results:**

Among 1860 patients, 453 (24.4%) achieved high ERP compliance. After matching, 437 well-balanced pairs (*n* = 874) were analyzed. The 12-month OS did not differ significantly between the high- and low-compliance groups before (95% vs. 92%; *p* = 0.081) or after (96% vs. 92%; *p* = 0.11) matching. In the matched cohort, high ERP compliance was not independently associated with 12 month mortality (adjusted hazard ratio, 0.68; 95% confidence interval, 0.36–1.30; *p* = 0.20). Long-term mortality was independently associated with metastatic disease, operative duration longer than180 min, and intraoperative hypotension. In subgroup analyses after matching, improved survival with high compliance was observed only for patients with colorectal liver metastases (*p* = 0.029).

**Conclusion:**

High ERP compliance reduces perioperative morbidity but is not independently associated with 12 month or long-term survival after LS.

**Supplementary Information:**

The online version contains supplementary material available at 10.1245/s10434-026-19459-7.

Although there have been notable advancements in the management of patients undergoing liver surgery (LS),^1,2^ postoperative morbidity impacts long term survival.^3–7^ The enhanced recovery program (ERP) is an evidence-based multimodal care program developed to minimize the response to surgical stress,^8–10^ with updated recommendations focusing on LS in 2022.^11^ Studies exploring the impact of ERP in LS suggest a strong dose-response relationship between high compliance with the protocol and improved outcomes, including morbidity,^12–15^ even when stratified for LS complexity.^12^

Although high ERP compliance may improve short-term outcomes, current evidence is inconclusive regarding its effect on overall and cancer-specific survival. Some studies in colorectal surgery suggest a potential survival benefit,^16–18^ whereas others do not,^19^ and this trend has not been clearly observed in LS.^20,21^ Because ERP is primarily designed to optimize early postoperative recovery, any survival effect, if present, is most likely to manifest within the first 12 months after LS, making 12 month survival a more relevant and sensitive endpoint for evaluating the influence of ERP compliance.

The current study was therefore designed to assess the association between ERP compliance and 12 month survival in a large, multicenter cohort of patients undergoing LS.

## Methods

### Study Design

The PRESTIGE (erP incReasE Survival afTer lIver surGEry) study was an open-label, prospective, European multicenter cohort designed to explore survival after LS in a target population of patients exposed to an enhanced recovery program (ERP).

### Participating Centers

The following 10 centers from France (FR), Switzerland (CH), and Belgium (BE) participated in the study: University Hospital of Besançon (FR), University Hospital of Dijon (FR), University Hospital of Lyon (unicancer) (FR), University Hospital of Lyon Sud (FR), University Hospital Henri Mondor (Créteil) (FR), University Hospital of Marseille (FR), Institut Curie (Paris) (FR), University Hospital of Liège (BE), University Hospital of Lugano (CH), and University Hospital of Toulouse (FR). Centers were eligible if they were affiliated with either the Enhanced Recovery After Surgery (ERAS) Society or the Francophone Group for Enhanced Recovery After Surgery (GRACE). Institutions not formally affiliated but implementing an ERP consistent with ERAS recommendations for LS^11^ also were included.

The study was structured and reported in accordance with the Strengthening the Reporting of Observational Studies in Epidemiology (STROBE) statement^22^ and the Reporting on ERAS Compliance, Outcomes, and Elements Research (RECOVER) checklist,^23^ ensuring transparent and standardized reporting of cohort studies and ERP-related outcomes.

### Patient Population

The study included adult patients (age > 18 years) who underwent elective LS in participating centers between June 2016 and November 2024. The ERP implemented in each institution followed a multimodal approach encompassing pre-, intra-, and postoperative elements of care consistent with ERAS recommendations for LS.^10,11^ The study excluded emergency procedures and patients with missing survival data.

### Data Source and Collection

Clinical data were prospectively collected using the GRACE-Audit platform (www.grace-audit.fr) or the ERAS-Audit system (https://encare.net/login), both adapted to LS guidelines and designed as interactive quality-improvement tools. For each patient, approximately 180 variables covering demographics, comorbidities, surgical characteristics, perioperative management, complications, and follow-up evaluation were recorded. Liver surgery was categorized into three complexity classes according to the Institut Mutualiste Montsouris (IMM) classification^24,25^: class I (wedge resection and left lateral sectionectomy), class II (anterolateral segmentectomy and left hepatectomy), and class III (posterosuperior segmentectomy, right posterior sectionectomy, right hepatectomy, central hepatectomy, and extended left/right hepatectomy. Data on overall and recurrence-free survival were obtained from institutional records and verified by the investigators of each center. To ensure encryption of patient data, a de-identified study number was assigned to each patient.

### Variables of Interest

*Compliance.* Overall ERP compliance was calculated as the proportion of fulfilled items among 21 ERP elements for each patient. Following prior studies in colorectal^17,26^ and liver surgery,^13,14^ high compliance was defined as ≥15 fulfilled items (≥ 70%).

*Survival.* Survival at 12 months was defined by censoring patients according to their survival status 12 months after LS. Overall survival (OS) was defined as the time from LS to death from any cause or the last follow-up visit, with censoring of patients alive at the end of follow-up period.

### Comparison

Patients achieving high ERP compliance ( ≥ 70%) were compared with those who had low ERP compliance ( < 70%). To minimize selection bias, patients with high ERP compliance were compared on a 1:1-matched low ERP-compliance control group based on preoperative variables.

### Outcomes

The primary outcome was 12 month OS after LS, estimated using the Kaplan–Meier method, comparing those who had high ERP compliance ( ≥ 70%) with those who had low compliance ( < 70%).

Secondary outcomes included identification of predictors of 12 month survival, evaluation of long-term OS (up to 5 years) and predictors within the whole cohort, subgroup analyses of the nested cancer cohort including, and long-term survival according to ERP compliance for the overall cancer cohort and for each cancer subgroup.

### Statistical Analysis

*Descriptive Statistics.* All statistical analyses were performed using R software (version 4.0.2 or later; R Foundation for Statistical Computing, Vienna, Austria). Data management and visualization were performed using standard R packages. Continuous variables are expressed as mean ± standard deviation or median (interquartile range), and categorical variables are expressed as counts and percentages. Group comparisons were performed using Student’s *t* test or the Mann–Whitney *U* test for continuous variables and the chi-square or Fisher’s exact test for categorical variables.

*Propensity Score-Matching.* To minimize selection bias and ensure comparability between groups, a propensity score-matching (PSM) analysis was performed using the MatchIt package. This approach was used to account for potential confounding factors by balancing the distribution of baseline characteristics between patients with high compliance (> 70%) and those with low compliance (< 70%).

*Model Specification.* The propensity score was calculated for each patient using a multivariable logistic regression model. The covariates included in the model were selected based on observed imbalances at baseline as well as clinical relevance and included age class, sex, American Society of Anesthesiologists (ASA) physical status, immune status (IMM), and cancer type.

*Matching Protocol.* Patients were matched using a 1:1 nearest-neighbor algorithm without replacement. A caliper of 0.2 of the standard deviation of the logit of the propensity score was applied to ensure the quality of the matches.

*Balance Diagnostics.* Post-matching balance was assessed using standardized mean differences (SMDs). A threshold of SMD smaller than 0.1 was used to define negligible imbalance for all included covariates. The overlap of the propensity score distribution (common support) was further verified through visual inspection of density and jitter plots. After the matching procedure, outcomes were compared in the matched cohort.

*Survival Analyses.* Analysis of 12 month survival was performed using Cox proportional hazards models, reporting hazard ratios (HRs) with corresponding 95% confidence intervals (Cis). Long-term OS was likewise evaluated with Cox models. Variables with a *p* value lower than 0.10 in univariable analyses or considered clinically relevant were entered into multivariable models. The graphic expression of survival overall is shown by the Kaplan-Meier curves.

Subgroup analyses were performed according to type of cancer. Within each subgroup, 12 month survival rates were compared between compliance groups using logistic regression, and Kaplan-Meier curves were generated to evaluate long-term survival, with log-rank tests used for group comparisons. An additional Cox model including centre_id as a covariate was performed to account for potential inter-center variability. A two-sided *p* value smaller than 0.05 was considered statistically significant.

## Results

During the study period, 1860 patients undergoing elective LS across 10 centers were included. The low-compliance group (< 70%) consisted of 1407 (75.6%) individuals, whereas the high-compliance group (> 70%) included 453 (24.4%) individuals. Missing data were present for less than 1% of the primary covariates.

### Descriptive Data and Matching Success

Baseline characteristics before and after matching are presented in Table 1. In the pre-matching cohort, significant differences were observed regarding age (63.23 ± 26.75 vs. 59.84 ± 14.11 years; *p* = 0.002), male sex (59.6% vs. 48.8%; *p* < 0.001), ASA score III or IV (34.4% vs. 20.3%; *p* < 0.001), disease type (e.g., colorectal liver metastasis [CRLM]: 36.0% vs. 29.1%; *p* < 0.001), and high surgical difficulty (IMM class III: 25.2% vs. 11.5%; *p* < 0.001).

**Table 1:** Patient and procedural characteristics before and after matching

	Before matching				After matching			
	ERP protocol compliance				ERP protocol compliance			
	Overall	<70%	>70%		Overall	<70%	>70%	
	(*n* = 1860) *n* (%)	(*n* = 1407) *n* (%)	(*n* = 453) *n* (%)	*p* Value	(*n* = 874) *n* (%)	(*n* = 437) *n* (%)	(*n* = 437 *n* (%))	*p* Value
Mean age (years, ± SD) ^a^	63.23 ± 26.75	64.32 ± 29.62	59.84 ± 14.11	0.002	61.67 ± 36.71	63.46 ± 49.92	59.89 ± 14.12	0.150
*Age (years)*								
<50	319 (17.2)	221 (15.7)	98 (21.6)	0.001	181 (20.7)	86 (19.7)	95 (21.7)	0.754
50–70	961 (51.7)	717 (51.0)	244 (53.9)		472 (54.0)	239 (54.7)	233 (53.3)	
>70	577 (31.0)	466 (33.1)	111 (24.5)		221 (25.3)	112 (25.6)	109 (24.9)	
Male sex	1060 (57.0)	839 (59.6)	221 (48.8)	<0.001	418 (47.8)	201 (46.0)	217 (49.7)	0.31
BMI >30 kg/m^2^ = 1	348 (18.7)	260 (18.5)	88 (19.4)	0.747	171 (19.6)	86 (19.7)	85 (19.5)	0.864
*ASA*								
I	229 (12.3)	144 (10.2)	85 (18.8)	<0.001	152 (17.4)	74 (16.9)	78 (17.8)	0.952
II	1053 (56.6)	777 (55.2)	276 (60.9)		540 (61.8)	272 (62.2)	268 (61.3)	
III	560 (30.1)	471 (33.5)	89 (19.6)		177 (20.3)	89 (20.4)	88 (20.1)	
IV	15 (0.8)	12 ( 0.9)	3 ( 0.7)		5 (0.6)	2 ( 0.5)	3 ( 0.7)	
*Disease, detail*								
Benign	406 (21.8)	296 (21.0)	110 (24.3)	<0.001	218 (24.9)	111 (25.4)	107 (24.5)	0.992
CRLM	638 (34.3)	506 (36.0)	132 (29.1)		263 (30.1)	132 (30.2)	131 (30.0)	
NCLM	279 (15.0)	162 (11.5)	117 (25.8)		204 (23.3)	99 (22.7)	105 (24.0)	
HCC	376 (20.2)	295 (21.0)	81 (17.9)		163 (18.6)	82 (18.8)	81 (18.5)	
CC	144 (7.7)	131 (9.3)	13 (2.9)		26 (3.0)	13 (3.0)	13 (3.0)	
*Difficulty classfication (IMM)*								
I	799 (43.0)	536 (38.1)	263 (58.1)	<0.001	489 (55.9)	242 (55.4)	247 (56.5)	0.943
II	651 (35.0)	513 (36.5)	138 (30.5)		280 (32.0)	142 (32.5)	138 (31.6)	
III	407 (21.9)	355 (25.2)	52 (11.5)		105 (12.0)	53 (12.1)	52 (11.9)	
*Intraoperative characteristics*								
Surgical approach								
Open laparotomy	1106 (59.5)	955 (67.9)	151 (33.3)	<0.001	436 (49.9)	294 (67.3)	142 (32.5)	<0.001
Minimally invasive (laparoscopy)	549 (29.5)	330 (23.5)	219 (48.3)		313 (35.8)	100 (22.9)	213 (48.7)	
Minimally invasive (robotic)	205 (11.0)	122 (8.7)	83 (18.3)		125 (14.3)	43 ( 9.8)	82 (18.8)	
*Length of surgery (min)*								
<90	546 (29.4)	354 (25.2)	192 (42.4)	<0.001	316 (36.2)	133 (30.4)	183 (41.9)	<0.001
90–180	1126 (60.5)	945 (67.2)	181 (40.0)		426 (48.7)	248 (56.8)	178 (40.7)	
>180	47 (2.5)	44 (3.1)	3 (0.7)		18 (2.1)	15 (3.4)	3 (0.7)	
*Intraoperative surgical complications*								
None	1317 (70.8)	881 (62.6)	436 (96.2)	<0.001	749 (85.7)	329 (75.3)	420 (96.1)	<0.001
Vascular injury	28 (1.5)	23 (1.6)	5 (1.1)		13 (1.5)	7 (1.6)	6 (1.4)	
Modification of surgical strategy	42 (2.3)	36 (2.6)	6 (1.3)		12 (1.4)	7 (1.6)	5 (1.1)	
Digestive tear	20 (1.1)	14 (1.0)	6 (1.3)		20 (2.3)	14 (3.2)	6 (1.4)	
*Intraoperative anesthesiologic complications*								
None	1324 (71.2)	898 (63.8)	426 (94.0)	<0.001	745 (85.2)	335 (76.7)	410 (93.8)	<0.001
Hypotension AND vasoconstrictors (P max <60 mmHg)	73 (3.9)	51 (3.6)	22 (4.9)		43 (4.9)	21 (4.8)	22 (5.0)	
Hypoxemia (SpO_2_ <92%)	10 (0.5)	5 (0.4)	5 (1.1)		6 (0.7)	1 (0.2)	5 (1.1)	
*Postoperative characteristics*								
Postoperative complications = 1	515 (27.7)	476 (33.8)	39 ( 8.6)	<0.001	146 (16.7)	109 (24.9)	37 ( 8.5)	<0.001
Death = 1	449 (24.1)	355 (25.2)	94 (20.8)	0.061	189 (21.6)	106 (24.3)	83 (19.0)	0.071
Death at 90 POD = 1	38 (2.0)	33 ( 2.3)	5 ( 1.1)	0.152	9 (1.0)	4 ( 0.9)	5 ( 1.1)	0.9
Mean OS (months ± SD)	27.78 ± 19.71	27.58 ± 20.67	28.41 ± 16.35	0.436	28.04 ± 18.81	27.82 ± 21.08	28.27 ± 16.24	0.728

ERP, Enhanced Recovery Program; BMI, body mass index; ASA, American Society of Anesthesiologists; CRLM, colorectal liver metastases; NCLM, non-colorectal liver metastases; HCC, hepatocellular carcinoma; CC, cholangiocarcinoma; IMM, Institut Mutualiste Montsouris (surgical complexity classification); POD, postoperative day; OS, overall survival; SpO_2_, peripheral oxygen saturation, SD standard deviation

After 1:1 propensity score-matching, 437 pairs (*n* = 874) were identified. An adequate balance across all preoperative covariates was observed, as indicated by the following standardized mean differences (SMD): age class (SMD, 0.051), sex (SMD, 0.073), body mass index (body mass index [BMI]: SMD, 0.037), ASA score (SMD, 0.039), disease type (SMD, 0.034), and surgical difficulty (IMM) (SMD, 0.023). In Fig. S1, SMD is resumed

### Outcome Data

In the matched cohort (*n* = 874), the high ERP compliance (>70%) group showed a higher rate of minimally invasive surgery (67.5% vs 32.7%; *p* < 0.001) and shorter operative times (*p* < 0.001). Intraoperative surgical complications occurred for 17 (3.9%) patients in the high ERP-compliance (>70%) group compared with 108 (24.7%) patients in the low ERP compliance (<70%) group (*p* < 0.001). Similarly, intraoperative anesthesiologic complications were reported in 27 (6.2%) and 102 (23.3%) patients, respectively (*p* < 0.001).

**Fig. 1:**
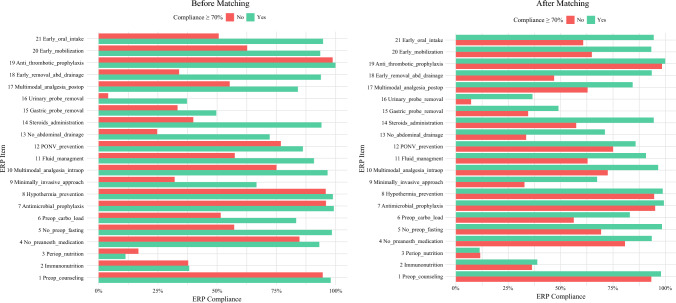
Enhanced recovery program (ERP) item compliance before and after matching

Postoperative complications were observed in 37 (8.5%) patients in the high ERP-compliance (>70%) group and 109 (24.9%) patients in the low ERP-compliance (<70%) group (*p* < 0.001). No significant difference was observed in 90 day mortality or mean OS. Patient and procedural characteristics are summarized in Table 1, and ERP item compliance is detailed in Fig. 1 and Table S1.

*Primary Outcome: 12 Month Survival After LS and ERP Compliance Before and After Matching.* In the overall cohort, the estimated 12 month survival was 95% (95% CI, 93–97%) among the patients with high ERP compliance and 92% (95% CI, 90–93%) among those with low compliance (*p* = 0.081). After matching, the estimated 12 month survival was 96% (95% CI, 93–98%) in the high-compliance group and 92% (95% CI, 90–95%) in the low-compliance group, with no statistically significant difference (*p* = 0.11).

*Secondary Outcomes: Predictors of 12 Month Mortality in the Matched Cohort.* The results of Cox regression analyses for predictors of 12 month mortality in the propensity score-matched cohort (*n* = 874) are summarized in Table 2.

**Table 2:** Uni- and multivariable Cox regression analyses for 12 month mortality in the matched cohort

	Univariate Analysis			Multivariate Analysis		
	HR	95% CI	*p* Value	HR	95% CI	*p* Value
*Age (years)*						
<50	–	–		–	–	
50–70	2.62	0.92–7.48	0.071	2.72	0.79–9.31	0.11
>70	2.84	0.93–8.62	0.066	3.6	0.99–13.1	0.052
*Sex*						
Female	–	–				
Male	1.04	0.58–1.85	0.9			
*BMI >30 kg/m*^*2*^						
0	–	–				
1	0.93	0.45–1.92	0.8			
*ASA*						
I	–	–				
II	1.65	0.64–4.23	0.3			
III	1.44	0.48–4.30	0.5			
IV	6.40	0.58–70.5	0.13			
*Disease, detail*						
Benign	–	–		–	–	
CRLM	2.07	0.82–5.21	0.12	1.94	0.63–5.96	0.2
NCLM	2.07	0.80–5.39	0.14	2.84	0.92–8.76	0.07
HCC	1.32	0.44–3.94	0.6	1.37	0.39–4.78	0.6
CC	1.08	0.13–9.01	>0.9	1.49	0.16–13.6	0.7
*Surgical complexity (IMM)*						
I	–	–				
II	1.62	0.86–3.03	0.13			
III	1.53	0.65–3.63	0.3			
*Length of surgery (min)*						
<90	–	–				
90–180	1.16	0.61–2.22	0.6			
>180	1.24	0.16–9.38	0.8			
*Intraoperative anesthesiologic complications*						
None	–	–		–	–	
Hypotension AND vasoconstrictors (P max <60 mmHg)	2.24	0.88–5.72	0.091	1.94	0.75–5.01	0.2
Hypoxemia (SpO_2_ <92%)	2.09	0.29–15.0	0.5	3.16	0.43–23.5	0.3
*Intraoperative surgical complications (%)*						
None	–	–				
Vascular injury	3.12	0.75–13.0	0.12			
Modification of surgical strategy	1.37	0.34–5.60	0.7			
Digestive tear	0.99	0.14–7.21	>0.9			
*Postoperative complications (%)*						
0	–	–		–	–	
1	2.05	1.08–3.89	0.029	1.66	0.79–3.50	0.2
*ERP protocol compliance (%)*						
<70	–	–		–	–	
>70	0.59	0.33–1.06	0.079	0.68	0.36–1.30	0.2

HR, hazard ratio; CI, confidence interval; BMI, body mass index; ASA, American Society of Anesthesiologists; CRLM, colorectal metastasis; NCLM, non-colorectal metastasis; HCC, hepatocellular carcinoma; CC, cholangiocarcinoma; IMM, Institut Mutualiste Montsouris (surgical complexity classification); SpO_2_, peripheral oxygen saturation; ERP, enhanced recovery program

In the univariable analysis, postoperative complications were significantly associated with increased 12 month mortality (hazard ratio [HR], 2.05; 95% CI, 1.08–3.89; *p* = 0.029). A trend toward higher mortality was observed for patients 50 to 70 years of age (HR, 2.62; 95% CI, 0.92–7.48; *p* = 0.071) and patients older than 70 years (HR, 2.84; 95% CI, 0.93–8.62; *p* = 0.066). Intraoperative hypotension requiring vasopressors also showed a non-significant association with mortality (HR, 2.24; 95% CI, 0.88–5.72; *p* = 0.091). High ERP compliance (≥ 70%) was associated with a non-significant trend toward reduced 12 month mortality (HR, 0.59; 95% CI, 0.33–1.06; *p* = 0.079). No significant associations were identified for sex, BMI class, ASA score, tumor type, surgical complexity (IMM class), duration of surgery, intraoperative anesthesiologic complications other than hypotension, or intraoperative surgical complications.

**Fig. 2:**
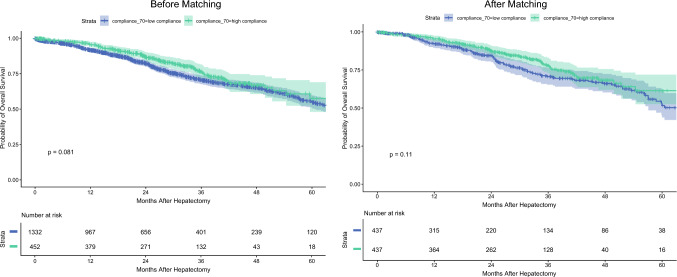
Kaplan–Meier analyses of long-term survival according to enhanced recovery program (ERP) compliance before and after matching

In the multivariable model, no variable reached formal statistical significance at the 0.05 threshold. Increasing age remained associated with a persistent trend toward higher mortality (age 50–70 years [HR, 2.72; 95% CI, 0.79–9.31; *p* = 0.11]; age >70 years [HR, 3.60; 95% CI, 0.99–13.1; *p* = 0.052]). Non-colorectal liver metastases showed a borderline association with increased mortality (HR, 2.84; 95% CI, 0.92–8.76; *p* = 0.07). After adjustment, postoperative complications were no longer independently associated with mortality (HR, 1.66; 95% CI, 0.79–3.50; *p* = 0.20). In the adjusted analysis, high ERP compliance was not independently associated with 12 month mortality (HR, 0.68; 95% CI, 0.36–1.30; *p* = 0.20). Similarly, sex, BMI class, tumor type, and intraoperative variables were not independently predictive of mortality in the multivariable model.

A sensitivity analysis including “Centre” as a covariate in the multivariable Cox model did not materially change the effect of high ERP compliance on 12 month mortality. Sensitivity analyses without centers or with additional covariates (postoperative complications, age, cancer type) yielded similar hazard ratios, suggesting that ERP adherence is associated with lower risk independently of center effects. The Cox regression analyses for predictors of 12 month mortality in the overall cohort are provided in Table S2.

*Long-Term OS in the Matched Cohort.* Long-term survival according to ERP compliance before and after matching is reported in Fig. 2. The results of Cox regression analyses for predictors of long-term mortality in the propensity score-matched cohort (*n* = 874) are summarized in Table 3. In the univariable analysis, older age, male sex, metastatic disease (both colorectal and non-colorectal), hepatocellular carcinoma, prolonged operative time ( > 180 min), and intraoperative hypotension requiring vasopressors all were associated with poorer long-term survival. High ERP compliance was not significantly associated with long-term survival (HR, 0.79; 95% CI, 0.59–1.05; *p* = 0.11).

**Table 3:** Uni- and multivariable Cox regression analyses for long-term survival in the matched cohort

	Univariate analysis			Multivariate analysis		
	HR	% CI	*p* Value	HR	95% CI	*p* Value
*Age (years)*						
< 50	–	–		–	–	
50–70	1.82	1.19–2.79	0.006	1.51	0.95–2.40	0.084
> 70	1.87	1.16–3.00	0.01	1.49	0.86–2.57	0.2
*Sex*						
Female	–	–		–	–	
Male	1.35	1.01–1.79	0.043	1.25	0.92–1.69	0.15
*BMI > 30 kg/m*^*2*^						
0	–	–				
1	0.94	0.65–1.35	0.7			
*ASA*						
I	–	–		–	–	
II	1.46	0.94–2.28	0.093	1.19	0.74–1.92	0.5
III	1.65	0.98–2.77	0.059	1.2	0.68–2.14	0.5
IV	1.44	0.34–6.15	0.6	0.79	0.11–6.00	0.8
*Disease, detail*						
Benign	–	–		–	–	
CRLM	3.47	2.02–5.93	<0.001	2.94	1.69–5.12	<0.001
NCLM	2.98	1.72–5.17	<0.001	2.98	1.70–5.21	<0.001
HCC	2.22	1.21–4.06	0.01	1.74	0.92–3.26	0.086
CC	1.36	0.40–4.67	0.6	1.15	0.33–4.03	0.8
*Surgical complexity (IMM)*						
I	–	–		–	–	
II	1.13	0.83–1.53	0.5	1.16	0.83–1.61	0.4
III	1.02	0.65–1.60	>0.9	0.98	0.61–1.59	0.9
*Length of surgery (min)*						
<90	–	–		–	–	
90–180	1.1	0.80–1.50	0.6	0.95	0.68–1.33	0.8
>180	2.98	1.29–6.90	0.011	2.64	1.08–6.47	0.033
*Intraoperative anesthesiologic complications*						
None	–	–		–	–	
Hypotension AND vasoconstrictors (P max <60 mmHg)	1.67	0.98–2.84	0.059	1.94	1.32–2.84	<0.001
Hypoxemia (SpO_2_ <92%)	0.45	0.06–3.24	0.4	0.68	0.09–4.87	0.7
*Intraoperative surgical complications (%)*						
None	–	–				
Vascular injury	0.96	0.24–3.89	>0.9			
Modification of surgical strategy	1.04	0.33–3.26	>0.9			
Digestive tear	1.15	0.47–2.82	0.8			
*Postoperative complications (%)*						
0	–	–		–	–	
1	1.37	0.97–1.94	0.07	1.33	0.92–1.90	0.12
*ERP protocol compliance (%)*						
<70	–	–		–	–	
>70	0.79	0.59–1.05	0.11	0.8	0.59–1.09	0.2

HR, hazard ratio; CI, confidence interval; BMI, body mass index; ASA, American Society of Anesthesiologists; CRLM, colorectal metastasis; NCLM, non-colorectal metastasis; HCC, hepatocellular carcinoma; CC, cholangiocarcinoma; IMM, Institut Mutualiste Montsouris (surgical complexity classification); SpO_2_, peripheral oxygen saturation; ERP, enhanced recovery program

In the multivariable analysis, the independent predictors of poorer long-term survival included colorectal liver metastases (HR, 2.94; 95% CI, 1.69–5.12; *p* < 0.001) and non-colorectal liver metastases (HR, 2.98; 95% CI, 1.70–5.21; *p* < 0.001). Operative duration longer than 180 min remained significantly associated with increased mortality (HR, 2.64; 95% CI, 1.08–6.47; *p* = 0.033), as did intraoperative hypotension requiring vasopressors (HR, 1.94; 95% CI, 1.32–2.84; *p* < 0.001). Increasing age showed a persistent but non-significant trend toward worse outcomes (age 50–70 years [HR, 1.51; 95% CI, 0.95–2.40; *p* = 0.084]; age >70 years [HR, 1.49; 95% CI, 0.86–2.57; *p* = 0.20]). After adjustment, postoperative complications were not independently associated with long-term survival (HR, 1.33; 95% CI, 0.92–1.90; *p* = 0.12). In the adjusted model, high ERP compliance was not independently associated with long-term survival (HR, 0.80; 95% CI, 0.59–1.09; *p* = 0.20). Analyses for the overall cohort are provided in Table S3.

*Subgroup Analyses of the Nested Cancer Cohort Before and After Matching.* In the nested cancer cohort of the overall population (77% of all patients), survival was 91% (95% CI, 89–93%) at 12 months, 67% (95% CI, 63–71%) at 36 months, and 51% (95% CI, 46–56%) 60 months in the low-compliance group compared with 95% (95% CI, 92–97%), 73% (95% CI, 67–79%), and 52% (95% CI, 42–65%), respectively, in the high-compliance group (*p* = 0.2, log-rank).

**Fig. 3:**
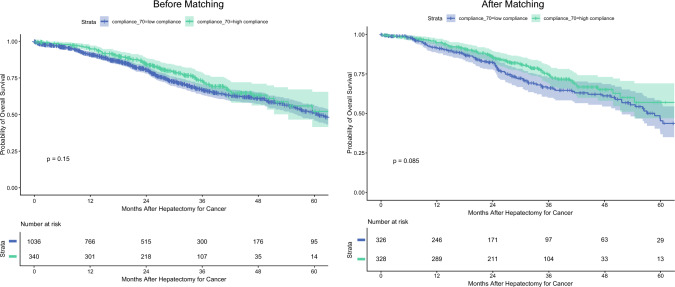
Kaplan–Meier analyses of long-term survival according to enhanced recovery program (ERP) compliance in the nested cancer cohort before and after matching

In the matched cohort, cancer patients represented 77.8% of the population (*n* = 680/874). Survival was 92% (95% CI, 88–95%) at 12 months, 66% (95% CI, 60–73%) at 36 months, and 45% (95% CI, 37–56%) at 60 months in the low-compliance group, versus 95% (95% CI, 92–97%), 75% (95% CI, 69–81%), and 57% (95% CI, 47–69%), respectively, in the high-compliance group (*p* = 0.085, log-rank; Fig. 3).

**Fig. 4:**
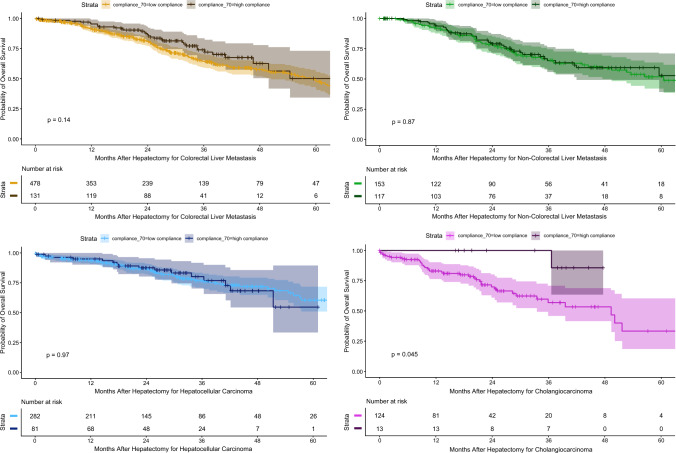
Kaplan–Meier analyses of long-term survival according to enhanced recovery program (ERP) compliance and cancer subgroup before matching

In subgroup analyses of the overall cohort, no significant differences were observed for CRLM (*p* = 0.14), NCRLM<AQ5> (*p* = 0.90), or hepatocellular carcinoma [HCC] (*p* > 0.90). In the patients with CC, survival was 83% (95% CI, 76–90%) at 12 months and 57% (95% CI, 46–71%) at 36 months in the low-compliance group compared with 100% at both time points in the high-compliance group (*p* = 0.045). Kaplan–Meier curves after matching by tumor type are presented in Fig. 4.

**Fig. 5:**
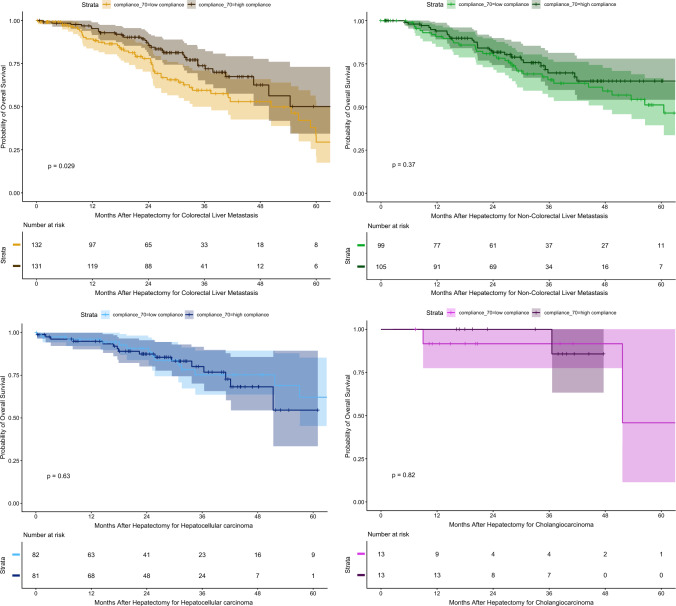
Kaplan–Meier analyses of long-term survival according to enhanced recovery program (ERP) compliance and cancer subgroup after matching

After matching, significant differences were observed only in patients who had CRLM, with survival of 89% (95% CI, 84–95%) at 12 months, 60% (95% CI, 50–71%) at 36 months, and 29% (95% CI,17–50%) at 60 months in the low-compliance group versus 95% (95% CI, 92–99%), 74% (95% CI, 65–84%), and 50% (95% CI, 34–73%), respectively, in the high-compliance group (*p* = 0.029). No significant differences were identified for NCRLM (*p* = 0.4), HCC (*p* = 0.6), or CC (*p* = 0.8). Kaplan–Meier curves after matching by tumor type are presented in Fig. 5.

## Discussion

In this multicenter cohort of patients undergoing elective LS, high ERP compliance was consistently associated with improved perioperative outcomes, including fewer intraoperative and postoperative complications after propensity score-matching. However, the primary objective of the study was not met because ERP compliance was not independently associated with 12 month survival, either before or after matching.

These findings reaffirm the well-established benefits of ERP in reducing perioperative morbidity and optimizing recovery, which remain clinically important regardless of effects on long-term survival. These observations align with prior literature on digestive and hepatic oncology, in which early studies suggested improved long-term outcomes with ERAS adherence,^16,17^ yet systematic reviews found inconsistent effects on survival. High ERAS adherence more reliably improved return to intended oncologic therapy (RIOT) and tolerance to adjuvant treatments, whereas direct effects on overall or disease-free survival remain uncertain. In LS, meta-analytic data do not support a mortality difference between ERAS and conventional pathways,^13^ reinforcing that benefits are primarily reflected in reduced morbidity and faster recovery.^15^

Secondary analyses further confirmed that none of the variables captured in the database independently predicted 12 month mortality in the matched cohort. In contrast, long-term survival was predominantly determined by intrinsic patient and disease characteristics as well as by procedural severity, with metastatic disease, prolonged operative time, and intraoperative hypotension requiring vasopressors emerging as independent predictors. These observations align with published literature suggesting that long-term outcomes largely reflect patient selection and procedural factors rather than the direct effect of ERP adherence.^1,12,15,27,28^

Subgroup analyses showed that the association between ERP compliance and survival varied by tumor type. Before matching, the most pronounced effect was observed in cholangiocarcinoma patients, with 12 and 36 month survival reaching 100% in the high-compliance group compared with 83 and 57%, respectively, in the low-compliance group. This pattern may reflect the higher baseline surgical risk and complexity of cholangiocarcinoma, in which structured perioperative care has greater impact.^29,30^ However, in the current study, this association concerned only 13 patients in the high-compliance group compared with 124 in the low-compliance group, and more importantly was no longer observed after matching. The very small size of this subgroup raises concern for potential type 1 error, and these findings should therefore be considered hypothesis-generating rather than confirmatory.

For the patients with CRLM, a survival difference favoring high ERP compliance persisted after matching, in contrast to other tumor types. This finding, although exploratory, may reflect the particular oncologic context of CRLM, in which postoperative recovery^31^ plays a critical role in timely resumption or initiation of systemic therapy (RIOT). Faster functional recovery and reduced complication burden may facilitate earlier adjuvant treatment, which is a key determinant of long-term outcomes in metastatic colorectal cancer. However, given the multiplicity of subgroup analyses and the absence of detailed data on perioperative or adjuvant chemotherapy, this association cannot be interpreted as causal and warrants confirmation in prospective, disease-specific studies.

The reasons why ERP compliance might appear beneficial in specific tumor types remain unclear and may reflect chance findings or unmeasured confounding. These findings overall indicate that the relationship between postoperative complications and long-term survival deserves further exploration in patients with colorectal liver metastases, particularly to understand how ERP compliance may influence this association.

Several limitations of this study should be acknowledged. The observational design entailed an inherent risk of selection bias because patients achieving high ERP compliance differed systematically from those with lower compliance in terms of age, comorbidity burden, disease type, and surgical complexity in the unmatched cohort. Propensity score-matching was applied to reduce this imbalance and achieved good balance across measured preoperative variables. However, residual confounding from unmeasured factors, such as frailty, nutritional status, liver function, or center-level organizational characteristics, cannot be excluded. A composite score was used to assess ERP compliance, precluding evaluation of the relative contribution of individual ERP components. Moreover, the database did not allow distinction between true patient- or provider-related non-compliance and clinically justified deviations due to complications or contraindications, which may represent different mechanisms and introduce potential reverse causality.

Statistical power was limited by the relatively small number of early mortality events, increasing imprecision in the identification of independent predictors of 12 month survival. The absence of detailed information on perioperative and adjuvant chemotherapy further constrains interpretation of long-term outcomes and precludes assessment of return to intended oncologic therapy (RIOT).

In addition, the relevance of RIOT as a unified endpoint is limited by marked heterogeneity across disease types and institutional practices. Adjuvant chemotherapy is commonly used after LS for metachronous colorectal liver metastases in some countries but not others. Systemic strategies for non-colorectal liver metastases are highly variable. No validated adjuvant therapy exists for hepatocellular carcinoma, and adjuvant capecitabine for cholangiocarcinoma is inconsistently applied depending on pathology and local practice.

The database also lacked key biologic parameters, major comorbidities such as cirrhosis, and procedure-specific postoperative complications (e.g., bile leak, ascites decompensation), all of which may have contributed to residual confounding. Subgroup analyses, particularly by tumor type, relied on small sample sizes, most notably for intrahepatic cholangiocarcinoma, raising the risk of type 1 error. Finally, the intrinsic heterogeneity of LS, encompassing diverse indications and operative strategies, may have diluted associations that could be more apparent in more homogeneous populations.

Despite these limitations, the large, multicenter cohort provided a comprehensive overview of real-world practice and supported the relevance of ERP adherence as an indicator of perioperative care quality in complex LS. Overall, these results reinforce that structured perioperative care optimizes recovery and reduces perioperative morbidity, even if it does not independently improve survival. Although intrinsic patient and disease factors remain the dominant determinants of survival, high ERP compliance serves as a global marker of perioperative care quality rather than a direct causal determinant of long-term survival. Maintaining high adherence may nonetheless enhance recovery trajectories and reduce complications, potentially creating favorable conditions for improved outcomes in selected high-risk subgroups.

Future prospective studies should aim to strengthen ERP adherence, assess the causal pathways linking perioperative optimization and outcomes, and explore specific benefits in subgroups such as colorectal liver metastases (earlier adjuvant therapy), hepatocellular carcinoma (reduced cirrhosis decompensation), and cholangiocarcinoma (lower postoperative morbidity).

## Conclusions

Although ERP compliance suggests improved short-term perioperative outcomes, it does not independently predict short- or long-term survival after LS. Importantly, these results do not challenge the current recommendation for ERP adherence, which remains central to enhancing recovery and perioperative outcomes.

## Supplementary Information

Below is the link to the electronic supplementary material.

Supplementary file1 Love plot compliance showing the distribution of standardized mean differences: comparison of preoperative characteristics between enhanced recovery program (ERP) compliance groups (>70 % vs <70 %) in the original and matched cohorts (JPG 459 KB)

Supplementary file2 (DOCX 49 KB)
