# Supplementary figures and images for: Enhanced Recovery After Liver Surgery: Does Compliance Impact Survival?
Source: Ann Surg Oncol. 2026 Apr 2;33(7):6076–89. doi: 10.1245/s10434-026-19459-7 (PMC13242497; doi:10.1245/s10434-026-19459-7)

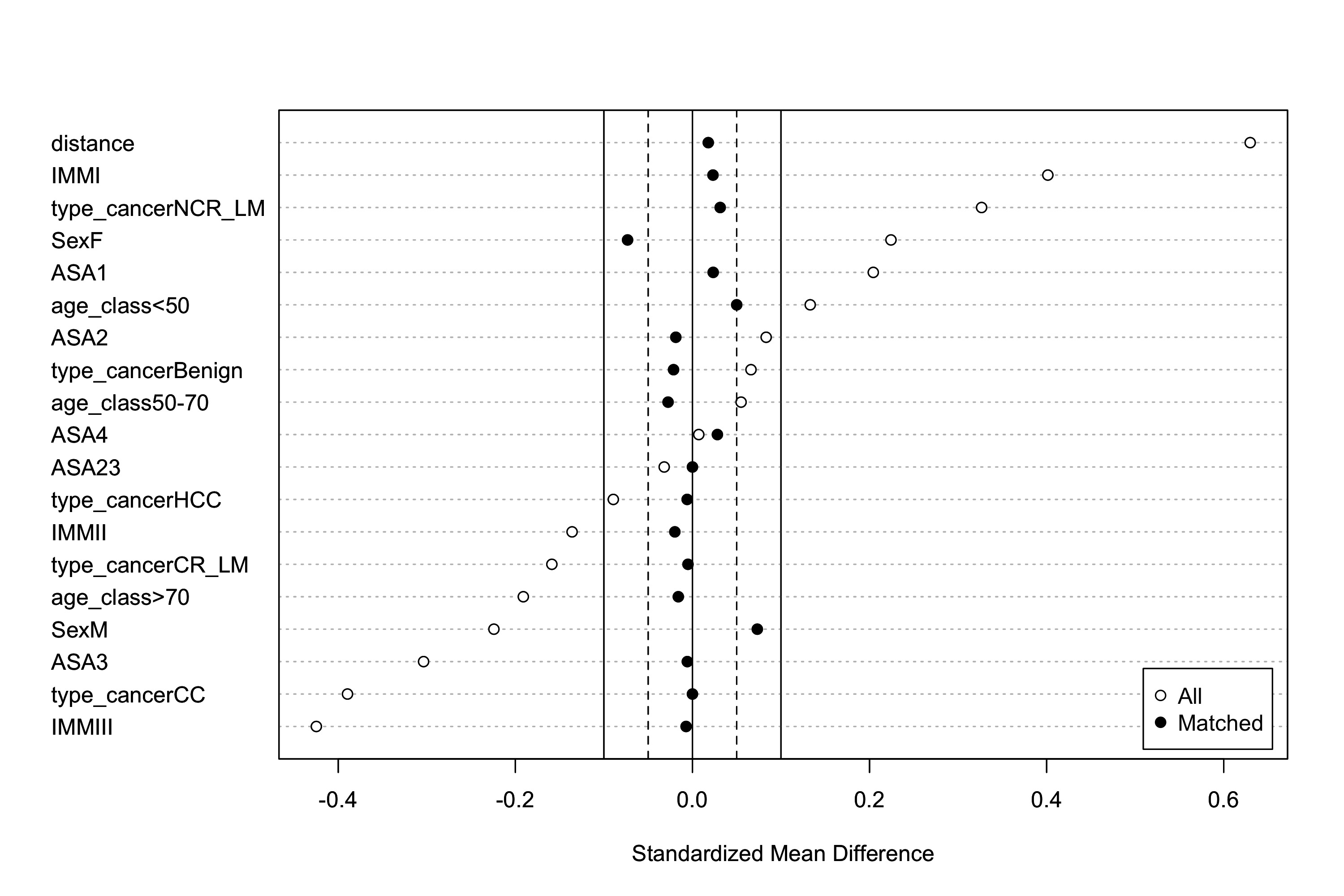

Supplement: Supplementary file 1 — Supplementary file1 Love plot compliance showing the distribution of standardized mean differences: comparison of preoperative characteristics between enhanced recovery program (ERP) compliance groups (>70 % vs <70 %) in the original and matched cohorts (JPG 459 KB) [file 10434_2026_19459_MOESM1_ESM.jpg]
