# Supplementary material for: Enhanced Recovery After Liver Surgery: Does Compliance Impact Survival?
Source: Ann Surg Oncol. 2026 Apr 2;33(7):6076–89. doi: 10.1245/s10434-026-19459-7 (PMC13242497; doi:10.1245/s10434-026-19459-7)
Supplement: Supplementary file 2 — Supplementary file2 (DOCX 49 KB) [file 10434_2026_19459_MOESM2_ESM.docx]

| **Supplementary Table 1 Enhanced recovery program (ERP) item compliance before and after matching** | | | | | | | | | |  | |  |  |
| --- | --- | --- | --- | --- | --- | --- | --- | --- | --- | --- | --- | --- | --- |
|  | **BEFORE MATCHING** | | | |  | | **AFTER MATCHING** | | |  | |  |  |
|  |  | **ERP Protocol Compliance** | |  | |  | | **ERP Protocol Compliance** | | |  |  |  |
|  | **Overall** | **<70%** | **>70%** |  | | **Overall** | | **<70%** | **>70%** | |  |  |  |
|  | **(n=1860)** | **(n=1407)** | **(n=453)** | **p** | | **(n=874)** | | **(n=437)** | **(n=437)** | | **p** |  |  |
| 1 Preop_counseling (%) | 1776 (95.5) | 1332 ( 94.7) | 444 ( 98.0) | 0.004 | | 836 (95.7) | | 408 (93.4) | 428 (97.9) | | 0.002 |  |  |
| 2 Immunonutrition (%) | 705 (37.9) | 532 ( 37.8) | 173 ( 38.2) | 0.929 | | 329 (37.6) | | 159 (36.4) | 170 (38.9) | | 0.485 |  |  |
| 3 Periop_nutrition (%) | 288 (15.5) | 237 ( 16.8) | 51 ( 11.3) | 0.005 | | 101 (11.6) | | 51 (11.7) | 50 (11.4) | | 1.000 |  |  |
| 4 No_preanesth_medication (%) | 1616 (86.9) | 1194 ( 84.9) | 422 ( 93.2) | <0.001 | | 762 (87.2) | | 353 (80.8) | 409 (93.6) | | <0.001 |  |  |
| 5 No_preop_fasting (%) | 1251 (67.3) | 805 ( 57.2) | 446 ( 98.5) | <0.001 | | 733 (83.9) | | 303 (69.3) | 430 (98.4) | | <0.001 |  |  |
| 6 Preop_carbo_load (%) | 1103 (59.3) | 725 ( 51.5) | 378 ( 83.4) | <0.001 | | 609 (69.7) | | 246 (56.3) | 363 (83.1) | | <0.001 |  |  |
| 7 Antimicrobial_prophylaxis (%) | 1800 (96.8) | 1350 ( 95.9) | 450 ( 99.3) | 0.001 | | 850 (97.3) | | 416 (95.2) | 434 (99.3) | | <0.001 |  |  |
| 8 Hypothermia_prevention (%) | 1797 (96.6) | 1349 ( 95.9) | 448 ( 98.9) | 0.003 | | 846 (96.8) | | 414 (94.7) | 432 (98.9) | | 0.001 |  |  |
| 9 Minimally_invasive_approach (%) | 754 (40.5) | 452 ( 32.1) | 302 ( 66.7) | <0.001 | | 438 (50.1) | | 143 (32.7) | 295 (67.5) | | <0.001 |  |  |
| 10 Multimodal_analgesia_intraop (%) | 1472 (79.1) | 1034 ( 73.5) | 438 ( 96.7) | <0.001 | | 739 (84.6) | | 317 (72.5) | 422 (96.6) | | <0.001 |  |  |
| 11 Fluid_managment (%) | 1220 (65.6) | 808 ( 57.4) | 412 ( 90.9) | <0.001 | | 672 (76.9) | | 275 (62.9) | 397 (90.8) | | <0.001 |  |  |
| 12 PONV_prevention (%) | 1474 (79.2) | 1083 ( 77.0) | 391 ( 86.3) | <0.001 | | 703 (80.4) | | 328 (75.1) | 375 (85.8) | | <0.001 |  |  |
| 13 No_abdominal_drainage (%) | 674 (36.2) | 347 ( 24.7) | 327 ( 72.2) | <0.001 | | 458 (52.4) | | 147 (33.6) | 311 (71.2) | | <0.001 |  |  |
| 14 Steroids_administration (%) | 988 (53.1) | 562 ( 39.9) | 426 ( 94.0) | <0.001 | | 664 (76.0) | | 251 (57.4) | 413 (94.5) | | <0.001 |  |  |
| 15 Gastric_probe_removal (%) | 694 (37.3) | 469 ( 33.3) | 225 ( 49.7) | <0.001 | | 365 (41.8) | | 151 (34.6) | 214 (49.0) | | <0.001 |  |  |
| 16 Urinary_probe_removal (%) | 225 (12.1) | 56 ( 4.0) | 169 ( 37.3) | <0.001 | | 192 (22.0) | | 32 (7.3) | 160 (36.6) | | <0.001 |  |  |
| 17 Multimodal_analgesia_postop (%) | 1158 (62.3) | 777 ( 55.2) | 381 ( 84.1) | <0.001 | | 644 (73.7) | | 275 (62.9) | 369 (84.4) | | <0.001 |  |  |
| 18 Early_removal_abd_drainage (%) | 877 (47.2) | 453 ( 32.2) | 424 ( 93.6) | <0.001 | | 614 (70.3) | | 205 (46.9) | 409 (93.6) | | <0.001 |  |  |
| 19 Anti_thrombotic_prophylaxis (%) | 1843 (99.1) | 1390 ( 98.8) | 453 (100.0) | 0.039 | | 867 (99.2) | | 430 (98.4) | 437 (100.0) | | 0.023 |  |  |
| 20 Early_mobilization (%) | 1305 (70.2) | 881 ( 62.6) | 424 ( 93.6) | <0.001 | | 692 (79.2) | | 284 (65.0) | 408 (93.4) | | <0.001 |  |  |
| 21 Early_oral_intake (%) | 1141 (61.3) | 712 ( 50.6) | 429 ( 94.7) | <0.001 | | 679 (77.7) | | 266 (60.9) | 413 (94.5) | | <0.001 |  |  |

| **Supplementary Table 2, Univariable and multivariable Cox regression analyses for 12-month mortality – whole cohort before matching** | | | | | | |  |
| --- | --- | --- | --- | --- | --- | --- | --- |
|  |  | **Univariate Analysis** | |  |  | **Multivariate Analysis** | |
|  | **HR***^1^* | **95% CI***^1^* | **p-value** |  | **HR***^1^* | **95% CI***^1^* | **p-value** |
| **Age** |  |  |  |  |  |  |  |
| <50 | — | — |  |  | — | — |  |
| 50-70 | 3.01 | 1.19, 7.60 | 0.020 |  | 2.48 | 0.95, 6.47 | 0.063 |
| >70 | 4.47 | 1.76, 11.4 | 0.002 |  | 3.74 | 1.40, 10.0 | 0.009 |
| **Sex** |  |  |  |  |  |  |  |
| F | — | — |  |  |  |  |  |
| M | 1.44 | 0.92, 2.26 | 0.11 |  |  |  |  |
| **BMI>30 Kg/m2** |  |  |  |  |  |  |  |
| 0 | — | — |  |  |  |  |  |
| 1 | 0.86 | 0.49, 1.53 | 0.6 |  |  |  |  |
| **ASA** |  |  |  |  |  |  |  |
| I | — | — |  |  | — | — |  |
| II | 5.20 | 1.27, 21.4 | 0.022 |  | 2.95 | 0.70, 12.5 | 0.14 |
| III | 5.76 | 1.38, 24.1 | 0.016 |  | 2.48 | 0.56, 11.0 | 0.2 |
| IV | 6.40 | 0.58, 70.5 | 0.13 |  | 2.99 | 0.26, 34.9 | 0.4 |
| **Disease, detail** |  |  |  |  |  |  |  |
| Benign | — | — |  |  | — | — |  |
| Colorectal metastasis (CRLM) | 1.39 | 0.66, 2.93 | 0.4 |  | 1.15 | 0.54, 2.44 | 0.7 |
| Non-colorectal metastasis (NCLM) | 2.14 | 1.0, 4.60 | 0.052 |  | 2.46 | 1.13, 5.37 | 0.023 |
| Hepatocellular carcinoma (HCC) | 1.59 | 0.73, 3.48 | 0.2 |  | 1.15 | 0.52, 2.58 | 0.7 |
| Cholangiocarcinoma (CC) | 4.57 | 2.05, 10.2 | <0.001 |  | 2.56 | 1.11, 5.88 | 0.027 |
| **Surgical Complexity (IMM)** |  |  |  |  |  |  |  |
| I | — | — |  |  | — | — |  |
| II | 1.35 | 0.81, 2.27 | 0.3 |  | 1.20 | 0.71, 2.04 | 0.5 |
| III | 2.29 | 1.36, 3.84 | 0.002 |  | 1.79 | 1.03, 3.12 | 0.040 |
| **Length of surgery** |  |  |  |  |  |  |  |
| <90 minutes | — | — |  |  |  |  |  |
| 90<minutes<180 | 0.95 | 0.38, 2.36 | >0.9 |  |  |  |  |
| >180 minutes | 1.27 | 0.55, 2.94 | 0.6 |  |  |  |  |
| **Intraoperative anesthesiological complications** |  |  |  |  |  |  |  |
| None | — | — |  |  | — | — |  |
| Hypotension AND vasoconstrictors (P max < 60 mmHg) | 2.63 | 1.40, 4.96 | 0.003 |  | 1.94 | 1.01, 3.75 | 0.048 |
| Hypoxemia (SpO2 < 92%) | 2.09 | 0.29, 15.0 | 0.5 |  | 3.16 | 0.43, 23.5 | 0.3 |
| **Intraoperative surgical complications (%)** |  |  |  |  |  |  |  |
| None | — | — |  |  |  |  |  |
| Vascular injury | 1.63 | 0.40, 6.64 | 0.5 |  |  |  |  |
| Modification of surgical strategy | 1.37 | 0.34, 5.60 | 0.7 |  |  |  |  |
| Digestive tear | 1.72 | 0.63, 4.71 | 0.3 |  |  |  |  |
| **Postoperative complications (%)** |  |  |  |  |  |  |  |
| 0 | — | — |  |  | — | — |  |
| 1 | 2.32 | 1.50, 3.60 | <0.001 |  | 1.77 | 1.09, 2.87 | 0.021 |
| **ERP Protocol compliance** |  |  |  |  |  |  |  |
| <70% | — | — |  |  | — | — |  |
| >70% | 0.51 | 0.31, 0.87 | 0.012 |  | 0.66 | 0.38, 1.15 | 0.14 |
| ERP = enhanced recovery program, HR = Hazard Ratio, CI = Confidence Interval, BMI = Body Mass Index, IMM = Institut Mutualiste Montsouris (surgical complexity classification), ASA = American Society of Anesthesiologists; | | | | | | | |

| **Supplementary Table 3, Univariable and multivariable Cox regression analyses for long-term mortality in the whole cohort, before matching.** | | | | | | |  | |
| --- | --- | --- | --- | --- | --- | --- | --- | --- |
|  |  | **Univariate Analysis** | |  |  | **Multivariate Analysis** | |  |
|  | **HR***^1^* | **95% CI***^1^* | **p-value** |  | **HR***^1^* | **95% CI***^1^* | **p-value** | |
| **Age** |  |  |  |  |  |  |  | |
| <50 | — | — |  |  | — | — |  | |
| 50-70 | 1.72 | 1.20, 2.46 | 0.003 |  | 1.52 | 1.04, 2.23 | 0.032 | |
| >70 | 2.10 | 1.44, 3.06 | <0.001 |  | 1.92 | 1.27, 2.91 | 0.002 | |
| **Sex** |  |  |  |  |  |  |  | |
| F | — | — |  |  | — | — |  | |
| M | 1.36 | 1.07, 1.71 | 0.011 |  | 1.30 | 1.02, 1.66 | 0.036 | |
| **BMI>30 Kg/m2** |  |  |  |  |  |  |  | |
| 0 | — | — |  |  |  |  |  | |
| 1 | 0.97 | 0.72, 1.29 | 0.8 |  |  |  |  | |
| **ASA** |  |  |  |  |  |  |  | |
| I | — | — |  |  | — | — |  | |
| II | 1.86 | 1.18, 2.93 | 0.007 |  | 1.44 | 0.90, 2.31 | 0.13 | |
| III | 2.10 | 1.31, 3.36 | 0.002 |  | 1.50 | 0.90, 2.51 | 0.12 | |
| IV | 1.44 | 0.34, 6.15 | 0.6 |  | 1.31 | 0.30, 5.76 | 0.7 | |
| **Disease, detail** |  |  |  |  |  |  |  | |
| Benign | — | — |  |  | — | — |  | |
| Colorectal metastasis (CRLM) | 2.17 | 1.45, 3.25 | <0.001 |  | 1.84 | 1.22, 2.78 | 0.004 | |
| Non-colorectal metastasis (NCLM) | 3.02 | 1.99, 4.60 | <0.001 |  | 3.41 | 2.22, 5.23 | <0.001 | |
| Hepatocellular carcinoma (HCC) | 1.82 | 1.16, 2.84 | 0.008 |  | 1.40 | 0.88, 2.21 | 0.2 | |
| Cholangiocarcinoma (CC) | 3.75 | 2.24, 6.29 | <0.001 |  | 2.85 | 1.67, 4.86 | <0.001 | |
| **Surgical Complexity (IMM)** |  |  |  |  |  |  |  | |
| I | — | — |  |  | — | — |  | |
| II | 1.01 | 0.77, 1.32 | >0.9 |  | 0.98 | 0.74, 1.28 | 0.9 | |
| III | 1.44 | 1.09, 1.91 | 0.011 |  | 1.35 | 1.00, 1.83 | 0.048 | |
| **Length of surgery** |  |  |  |  |  |  |  | |
| <90 minutes | — | — |  |  |  |  |  | |
| 90<minutes<180 | 0.99 | 0.62, 1.56 | >0.9 |  |  |  |  | |
| >180 minutes | 1.06 | 0.69, 1.63 | 0.8 |  |  |  |  | |
| **Intraoperative anesthesiological complications** |  |  |  |  |  |  |  | |
| None | — | — |  |  | — | — |  | |
| Hypotension AND vasoconstrictors (P max < 60 mmHg) | 1.88 | 1.28, 2.78 | 0.001 |  | 1.72 | 1.14, 2.59 | 0.010 | |
| Hypoxemia (SpO2 < 92%) | 0.45 | 0.06, 3.24 | 0.4 |  | 0.62 | 0.09, 4.51 | 0.6 | |
| **Intraoperative surgical complications (%)** |  |  |  |  |  |  |  | |
| None | — | — |  |  | — | — |  | |
| Vascular injury | 0.75 | 0.24, 2.36 | 0.6 |  | 0.61 | 0.19, 1.98 | 0.4 | |
| Modification of surgical strategy | 1.91 | 1.02, 3.59 | 0.045 |  | 1.46 | 0.75, 2.84 | 0.3 | |
| Digestive tear | 0.95 | 0.47, 1.92 | 0.9 |  | 1.09 | 0.54, 2.22 | 0.8 | |
| **Postoperative complications (%)** |  |  |  |  |  |  |  | |
| 0 | — | — |  |  | — | — |  | |
| 1 | 0.79 | 0.61, 1.01 | 0.057 |  | 0.83 | 0.64, 1.09 | 0.2 | |
| **ERP Protocol compliance** |  |  |  |  |  |  |  | |
| <70% | — | — |  |  | — | — |  | |
| >70% | 0.79 | 0.61, 1.01 | 0.057 |  | 0.83 | 0.64, 1.09 | 0.2 | |
| ERP = enhanced recovery program, HR = Hazard Ratio, CI = Confidence Interval, BMI = Body Mass Index, IMM = Institut Mutualiste Montsouris (surgical complexity classification), ASA = American Society of Anesthesiologists; | | | | | | | |  |
